# Hospital and Institutional News

**Published:** 1919-11-29

**Authors:** 


### HOSPITAL AND INSTITUTIONAL NEWS.

### SIR COOPER PERRY, M.A., M.D., F.R.C.P.

A very interesting announcement to most hospital and all University authorities was made in the *Times* of November 20. It recorded the appointment of Sir Cooper Perry, M.A., M.D., F.R.C.P., Superintendent and Consulting Physician to Guy's Hospital, and Vice-Chancellor of the University of London, by the Senate of the University to the post of its Principal Officer, which has been in abeyance since Sir Henry Miers' resignation in the summer of 1915. Sir Cooper Perry was educated at Eton and King's College, Cambridge. He gained the Bell, Browne and Pitt Scholarships and the Browne Medal. He graduated as Senior Classic in 1880, and was a Fellow of his College, 1880-87. Sir Cooper edited, with Dr. Marsh, the anthem-book still in use at King College, Cambridge. Having gained some of the most coveted of the scholastic honours at Cambridge, he looked round for a profession and embraced that of medicine. This entailed a further residence at Cambridge for several years, which he entered into with zest, and in due course became M.R.C.S.Eng. 1885, M.B.Camb. 1886, M.D. 1888, M.R.C.P.Lond. 1889, F.R.C.P.Lond. 1894. From Cambridge he went to the London Hospital, where he was resident house physician and house surgeon for twelve months. Thence he went to Guy's Hospital, where he was appointed assistant physician in 1887, Dean of the Medical School in 1888, and Warden of the College in 1890. In December 1892 Sir Cooper was appointed Superintendent of Guy's Hospital, when he retained the office of assistant physician, but relinquished those of Dean and Warden. He was then thirty-six, in the prime of his manhood, and covered with honours which he had gained by strenuous work and the exercise of a brain-power which few men possess and all must envy. The truth of this has been demonstrated in many fields since 1892. He became physician to the Skin Department at Guy's and was soon recognised as a master. He is the author, with Dr. Shaw, of "Malignant Diseases of the Stomach" (Guy's Hospital Reports), 1891, "Diseases of the Duodenum," *Ib.* 1893, and "Malignant Diseases of the Stomach," *Ib.* 1904. He has held important lectureships in Guy's Hospital Medical School, and examinerships in Materia Medica and Pharmacology at the London University, and in Materia Medica and Medicine under the Conjoint Board. He is an active member of Committee of the Evelina Hospital for Children and other important undertakings. He was appointed a member of the Distribution Committee of King Edward's Hospital Fund twelve years ago, and is at the present time an active and invaluable member of its Council. He rendered important services to the Government in Egypt in reorganising the Cairo Medical School in 1897, and was knighted in 1903 for his eminent services in connection with the reorganisation of the R.A.M.C. His acceptance of the new office entails his retirement from the Guy's Hospital superintendentship, which he has held for twenty-seven years. This service entitles him to a pension, which we have no doubt will be commensurate with the splendid services he has rendered to Guy's, which we deal with in an article on page 189 of this issue.

### THE HOSPITAL CRISIS.

Courage is returning, and hospitals, small and large, up and down the country are showing signs of life and resolution in facing the crisis which threatens the voluntary hospitals during the next three years. Liverpool, always alive and awake, is through the Lord Mayor raising £80,000 to provide a sum estimated to be about the amount which will be required to enable the voluntary hospitals in Liverpool to weather the war storm. At a meeting of the British Hospitals Association last week it was stated that Oxford's appeal is going forward with continuous developments, an important one being the resolution of the working classes to join hands with Mr. Cronshaw, the treasurer, and do their utmost to help raise the £50,000 required. This decision was come to after an interesting debate in which the extraordinary view of some members of the working-classes was expressed that all hospitals should go on the rates, and then the working-classes would have a good time. Did the less fortunate of their number have a good time in workhouses in the old days? Have German patients had a good time in the rate-supported hospitals of the German Empire, or in Austria and elsewhere? It is the work of a Good Samaritan who knows the facts to take every opportunity to enlighten the working classes on what rate-supported hospitals might mean in this country, and the possible fresh miseries and risks which they might incur under a system of hospitals where the old workhouse excuse for inefficiency would speedily re-appear. Every just cause of complaint, discomfort or inefficiency was liable to be met by the assertion that, whatever the treatment, it was good enough for a State hospital, good enough for a workhouse. Rate support has taught us that it too often means indiscipline, indifference to efficiency and the discomfort of those who most need and require the tenderest and kindest of treatment continuously. It is every hospital man's privilege to lose no opportunity to tell the truth, the whole truth and nothing but the truth when discussing hospital questions with those members of the community who hold that the rates, always and for everything, is the surest way to provide blessings for the humblest of the sick, and even for themselves and members of their family when ill. Those whom the gods desire to destroy they first drive mad.

### TWO RIVAL CITIES: NAME THE WINNER.

In The Hospital of November 8 we published a scheme whereby Glasgow could bring honour on that city and link its name for all time with Lord Lister, the great Life-Saver of Mankind. It was announced some three weeks ago that a Committee had been appointed to consider these proposals, but nothing further has been published since. Meanwhile, that other great city, Liverpool, which aspires too to be regarded as the second city in the Empire, has evidently taken the suggestion to heart and, headed by the Lord Mayor, strenuous efforts are being made to raise the sum of £80,000 to be held as security to meet the special requirements of the hospitals of Liverpool during the next three years, owing to after-war conditions. The sand is passing through the hour-glass. The most valuable time of the year for raising money may be lost by further inaction in the North. Liverpool, with its unique energy, may and probably will easily complete its £80,000, bank it by December 31, 1919, and so take the premier position amongst cities.

### SMALLER HOSPITALS.

The small communities throughout the country are actively interesting themselves in the provision of hospital accommodation and the maintenance and upkeep of the hospital beds on which they and their neighbours are dependent in the day of sickness. Stamford has selected for its war memorial the erection at the Stamford Infirmary of a children's ward of 12 beds in honour of all those glorious heroes from the town and district who gave their lives during the Great War 1914-1918 in the cause of right and freedom. In connection with this scheme considerable improvements in the hospital are contemplated, for which the money has been already provided. A maternity ward of ten beds costing £5,000, and the building of a nurses' home costing £6,000, with further material improvements costing £2,900, were also mentioned at a meeting presided over by the Mayor, Mr. W. E. Martin, held in the Town Hall. The management of the War Memorial was placed in the hands of a committee consisting of representatives from all sections of the community and from all parts of the town and district. It was hoped that the Red Cross Society would contribute £1,000. We are indebted to Miss M. Goodrich, the Matron, for the newspaper cutting conveying this interesting information.

### DERBY'S OPPORTUNITY.

The people of Derby have a great opportunity to help hospital and nursing work in that city. It has been determined to erect a new out-patients' department, and to build an extension to the nurses' home estimated to cost £50,000. Towards the sum required the County Directors of the Derbyshire Auxiliary Hospitals have offered £14,000; £14,000 more will be raised by the Governors of the Derbyshire Royal Infirmary, and the Infirmary Saturday Committee have raised £7,090, thus exceeding all previous records, the contribution from this Committee in 1914 being £2,921. Some inhabitants of Derby city, including Mr. G. Herbert Strutt, seem to be pessimistic about the financial future of the excellent voluntary hospitals Derbyshire people have so much to be thankful for. Let him study Liverpool's example and put himself at the head of a movement to raise an adequate sum to be funded as a hospital reserve for the three uncertain years of difficulty ahead, 1920-3. Then pessimism will die and hospital efficiency and the adequate treatment of the sick should be of the best in the city of Derby. It is a great satisfaction to find week by week that hospital people throughout the country are waking up to the true position of the future outlook of our voluntary hospitals. It is that a little courage and the aid of an organised system of friendly workers in every city and town throughout the country will secure adequate funds without difficulty, and the maintenance of all sick people who are dependent upon hospital care in the days of accident and illness.

### THE GENERAL MEDICAL COUNCIL ELECTION.

As was anticipated in these columns a short time ago, the four candidates selected for support by the British Medical Association secured election to the General Medical Council as direct representatives of the medical profession in England. Sir T. Jenner Verrall headed the poll with rather more than five thousand votes; and his three fellow nominees each received over four thousand. The candidates put forward by the Medico-Political Union made quite a good showing, however; Mr. W. F. H. Coke, the president of that body, received about three thousand six hundred votes, and the others not many less. The independent candidates received far fewer suffrages. The surprise of the election is the unexpected strength polled by the Medico-Political Union; and the British Medical Association will do well to bear this constantly in mind. If the Association ever puts forward candidates who are defeated the Association is politically dead thenceforward; and the managers of that Association will do well before the next election to pay rather more attention to the consensus of professional opinion (as far as it can ever be gauged, which is always difficult in so inarticulate a profession) than it has sometimes done in the past.

### THE RETIREMENT OF A SENIOR SURGEON.

Following an old established custom, a gathering of governors and past and present members of the staff of the Samaritan Hospital for Women, Marylebone, gave a complimentary dinner to Mr. J. D. Malcolm at the Imperial Restaurant on November 22, on the occasion of his retirement from the post of senior surgeon to the hospital after nearly thirty-six years of service to that institution. The custom was last observed, we believe, some years ago when Mr. Alban Doran retired; the years of war have naturally prevented such festivals during recent times. The chair was taken by the chairman of the committee, the Rev. A. W. Oxford, who has himself grown grey in the management of the Samaritan Hospital; and amongst those present was the treasurer, Mr. Nash. Both these gentlemen are said to have voted for Mr. Malcolm's election to the assistant staff all those years ago.

### Y.M.C.A. £600,000 DEFICIT.

The armistice found the great war charities in diverse position. The National Relief Fund had several millions which the executive sternly refuse to disgorge. The British Red Cross Society had managed its gigantic organisation so well that in winding-up, all liabilities could be met, and a substantial balance remain available for peace work of a corresponding nature. But the Young Men's Christian Association had not, or were unable to have, the foresight to cut its clothes according to the cloth available, with the result that there is at present a deficit of over £600,000, and a prospect of a debit balance at the end of the year of nearly three-quarters of a million. This situation was revealed at a luncheon given a few days ago to Sir Henry McMahon at the Cannon Street Hotel, when it was announced that though the Association was solvent at armistice-time, clearing up the many activities has resulted in the financial position stated. The public stopped contributions when the hostilities ceased and huge stores were on hand, many of them unsaleable, including tons of mincemeat for Christmas puddings for the troops. The Association need not be pessimistic about the mincemeat as, if it is eatable, the voluntary hospitals can take a considerable quantity. An appeal is being organised to meet the position and continue the work of the Association.

### "MOTHERS OF DISEASE."

In a paragraph appearing under this title, the *Times* has recently called attention to the fact that there is a serious increase of scarlet fever and diphtheria in London, and since this announcement, and the fact that, as Dr. Addison states it, there is "a substantial increase compared with the numbers of the past five years," would not alone perhaps attract such public attention as they deserve, we think that our contemporary raises, in his comments, a point which *must* be realised by the laity, particularly by those members who, by virtue of administrative capacity, exert an indirect influence on the nation's health measures. In both these diseases the ill-effects are by no means limited to the initial attack. In fact the public loss immediately due to the acute illness is of relatively low importance when compared with that arising from the remote effects of ill-health which may haunt the victim throughout his lifetime. It is another instance of the vast need and urgency of co-ordinated prevention and cure, and one of those essential points in the actual story of epidemic disease in this country which, when fully realised by the laity, may help indirectly to remove some of the financial difficulties under which most matters medical seem at the moment to be labouring.

### THE INFLUENZA REPORT.

It is more than impressive to read in the Medical Research Committee's Report just published that, since the armistice, influenza has killed more young people than fell on the battlefields throughout the war, and it is by no means to this disease alone that an appalling loss of precious English lives is due. It is well known how successfully an army of medical research organisations grappled with the terrible casualties of war, but it is probably not so well known that the great majority of them have since been disbanded because there is no money forthcoming to support them. These matters are indeed a national problem, and, though already frequently mentioned, cannot be referred to too often. In the case of the Committee itself it will, under the Ministry of Health Act, be controlled by a committee of the Privy Council, but, as its own report points out, unless the scientific worker is financially secure he will suffer from continual temptations to turn aside to professional practice or administrative work in which remuneration the worldly influence and, too often, the worldly recognition is so much higher. These temptations are fatal if they are not reinforced by economic compulsion.

### POLICEMAN OR DOCTOR?

There has been much amusing comment in the recent Press on the very obvious tendency, at the moment at least, for fortune to favour the non-professional classes. It is but a step further to the statement that, financially speaking, this is the age of the manual worker and not of the mental, and we heartily agree with a contemporary who before opening his true jest, suggests that parents of the professional classes have got to think along new lines where the careers for their boys are concerned. Proceeding, one is asked, however, to picture a boy, quite a good fellow, as intelligent as the average boy who has been to a public school but not more so, and whose parents are convinced that the police force is going to offer him better chances than any of the professions.* A comparison is then drawn, stage by stage, and true it is that figures and probabilities are found to favour the reasonably educated policeman! But surely we have not, in England, reached the phase which this light-hearted writer would suggest, when it is time to realise that for this generation brains are by way of being a back number and that brawn has come into its own. A poor result of a well-won war, but our present crowded medical schools disprove any general acceptance of so fatal a situation.

### HEALTH DISCUSSION IN LANCASHIRE.

At the recent meeting of members of the Royal Sanitary Institute at Bootle Town Hall a discussion took place upon the very central point of many problems of pressing urgency and importance—namely, the reconstruction of Public Health Administration. It is impossible to do more than select a few of the outstanding ideas circulated, but many of these are well worthy of attention. In the first place, it was pointed out that, although a highly important step towards unification had been made to the extent shown in the Ministry of Health Act of this year, yet complete unification, with all that it implies, is very far from being an accomplished fact. At present it is true that we have a new advantage of primary importance in administration in that one subject in its various phases is dealt with by one controlling body, but, while this is so for the high authority, there is still much to be done before duality of lesser and local control, conflict of authority and waste of energy, can be eradicated. Also the need for more reasoned medical understanding of the masses was repeatedly made apparent, and here indirectly arise innumerable difficulties from the fact that as yet the typical situation does not show one a family doctor who can carry the very principles of health into the people's homes. Later it was stated that no less a proportion than 20 per cent. of the persons who died of consumption in Lancashire were never notified to the Public Health authority. Thus the speakers passed on to notification itself, and its failings were fully discussed. The necessity for a propaganda department was strongly urged, and there is no doubt that non-notification of infectious disease occurs with distressing frequency through ignorance alone. On the whole, the subject before the meeting was of such magnitude that it was hardly to be expected that in so short a time more than the principles involved could be touched upon, but this and similar concerted attempts to understand and act in accordance with the needs of the present situation are deserving of all encouragement and sympathy.

### A NEW SIDELIGHT ON SWIFT.

At this season it is customary for the committee of St. Patrick Dun's Library to present its report to the Royal College of Physicians of Ireland. Notwithstanding the occupation of the Library by the Ministry of Health during almost the whole of the past year, the report is of unusual interest, for it gives the text of three letters written by the eminent Irish doctor, Sir Edward Barry, Bart., in 1737, to Lord Orrery, a peer who is remembered because he was known by Swift. Barry, who was born in Cork in 1698, followed the fashion of the time in studying medicine, under the learned Boerhaave at Leyden, where he graduated M.D. in 1719. By 1740 he had become President of the Queen's College of Physicians. Eventually his success led him to come to London, but according to Boswell he did not repeat it in the Metropolis. He was created a baronet, however, in 1775, after having settled in retirement at Bath, where he died a year later. He is interesting to us mainly for literary and historical reasons. He wrote a work upon "The Wines of the Ancients," and the better-known correspondence with Lord Orrery (published in 1913 in the "Orrery Papers") to which the three unpublished letters must be added. They contain interesting references to Swift, to a case of ossification and to the alleged beating of Swift by Wilson, whose sorry repute does not improve with the years. The report then is a triumph for a lean year of enforced inactivity, and we congratulate the registrar and the librarian on the publication of those letters.

### PROPHYLACTIC VACCINATION.

We have in these pages so frequently advocated the more general adoption of prophylactic vaccination measures against the innumerable catarrhs and their complications which are liable to assail the inhabitants of this country, and as frequently encountered an attitude of marked scepticism among both the lay and professional public. Thus it is with no little interest and approval that we note the recent announcement by Dr. Addison that the Ministry of Health are now making arrangements for the preparation and supply of "anti-influenza" vaccine to be made available to medical officers of health for distribution to all practitioners in their areas who desire it. The decision of authority may not be invariably the correct one, but nevertheless in this instance such an undertaking would not be made without very much more reasoned and favourable evidence of prophylactic value than some of the critics have in the past admitted to exist.

### SALARIES FOR SCHOOL MONITORS.

The reform of the Poor-Law system can hardly be studied at a better place than Birmingham, where every care is taken to keep children out of the workhouse and to guard them as they grow from any pauper taint. The cottage homes for the children, the education offered to promising talent, the apprenticeship given, and the after-care homes to which the apprentices return after their work, all these show that a public authority need not be a bad substitute for a good parent. In a recent report by Sir James Curtis a remarkable instance is quoted of a girl engaged by the Worcestershire Education Committee as monitor in the village school at Welford at a salary of £20 a year. This appointment will help her to continue her education while she is learning to teach, and when she reaches the age of sixteen she will be free to decide whether she would like to adopt teaching as her profession, or something else. Having begun to earn money so early at teaching, presumably she will continue her successful start, and it is interesting to reflect that few private parents could have won for her an equal opportunity so early.

### THIS WEEK'S DRUG MARKET.

It is remarkable how the upward tendencies of prices continues in face of the increasing supplies. In some cases this is due to the continuing demand from all parts of the world, but in other cases it is difficult to account for the upward movement. Among the synthetic drugs hexamine, phenazone, resorcin, acetanilide, and phenacetin are all inclined to move upwards in price. The value of bromides is fully maintained. English refined camphor is dearer. Chamomiles are more freely offered at rather lower rates. The good demand for eucalyptus oil continues. Formaldehyde is scarce and dearer. Menthol and Japanese peppermint oil are again dearer. Podophyllum root is dearer. Squill, senega root, and saffron tend upwards in price. At the recent public sale of drugs in Mincing Lane the demand was very fair. Aloes was dearer; the value of honey was maintained; full prices were paid for sarsaparilla; cascarilla bark was dearer; Cubebs fetched higher prices; senna sold at steady rates.

